# Transfusion Thresholds and Neurological Functional Outcome After Acute Brain Injury: An Updated Systematic Review and Meta-Analysis of Randomized Clinical Trials

**DOI:** 10.3390/jcm14103487

**Published:** 2025-05-16

**Authors:** Pierludovico Moro, Marco Andrighetti, Giovanni Siconolfi, Maria Sole Borioni, Carlo Di Bonaventura, Danilo Toni, Emanuele Cerulli Irelli

**Affiliations:** 1 Department of Human Neurosciences, Sapienza University, 00185 Rome, Italy; pierludovico.moro@uniroma1.it (P.M.); marco.andrighetti@uniroma1.it (M.A.); mariasole.borioni@uniroma1.it (M.S.B.); carlo.dibonaventura@uniroma1.it (C.D.B.); danilo.toni@uniroma1.it (D.T.); 2 Department of Neuroscience, Catholic University of the Sacred Heart, 00185 Rome, Italy; siconolfig@yahoo.it

**Keywords:** blood transfusion, Glasgow Outcome Scale, subarachnoid hemorrhage, traumatic brain injury, intensive care unit

## Abstract

**Background/Objectives**: The benefits of liberal transfusion strategies for neurological outcomes in critically ill patients with acute brain injuries (ABIs) remain uncertain due to conflicting evidence and potential risks. This study aimed to evaluate the efficacy and safety of a liberal transfusion strategy in adults with ABI. **Methods**: A systematic review of PubMed, Scopus, and the Cochrane Library was conducted from inception until 18 December 2024. Randomized clinical trials (RCTs) comparing liberal and restrictive transfusion strategies in adult patients admitted to intensive care units with ABI were included. The primary outcome was unfavorable neurological function at the last follow-up, defined as Glasgow Outcome Scale (GOS) score <4, Extended GOS score <5, or modified Rankin Scale score >3. **Results**: Among 5859 screened records, five RCTs (2385 patients) met the inclusion criteria. Liberal transfusion significantly reduced unfavorable neurological outcomes (RR, 0.88; 95% CI, 0.82–0.95; *p* = 0.0009) without affecting mortality (RR, 0.97; 95% CI, 0.84–1.11; *p* = 0.66). A meta-analysis of two studies (n = 1465 patients) showed improved functional independence with liberal strategies (MD, 6.70; 95% CI, 2.07–11.33; *p* = 0.005) but no difference in quality of life (*p* = 0.30). Sepsis or septic shock occurred less frequently in the liberal group (RR, 0.68; 95% CI, 0.50–0.92; *p* = 0.01). Subgroup analysis indicated that liberal strategies improved neurological outcome in traumatic brain injury (TBI) patients (RR, 0.89; 95% CI, 0.82–0.97; *p* = 0.01) but did not yield significant differences in spontaneous subarachnoid hemorrhage (*p* = 0.09). **Conclusions**: Liberal transfusion strategies safely improve neurological outcomes in adults with ABI, specifically in the subgroup of TBI, whereas further studies are needed in patients with SAH.

## 1. Introduction

The optimal hemoglobin concentration for critically ill patients with acute brain injury (ABI) remains a subject of ongoing debate. Anemia is associated with increased morbidity and mortality in this population, and a liberal transfusion strategy might theoretically provide benefits by enhancing oxygen delivery and mitigating brain tissue hypoxia, particularly in cases of impaired cerebral autoregulation or reduced cerebral blood flow [1,2]. While red blood cell (RBC) transfusion is commonly used to prevent or treat anemia in this population, uncertainty persists regarding the hemoglobin thresholds at which transfusion becomes beneficial or potentially harmful. Complications such as secondary infections, lung injury, and thromboembolic events underscore the need for a careful balance when determining transfusion thresholds [3,4]. This dilemma has driven extensive research aimed at identifying transfusion strategies that minimize harm while optimizing neurological outcomes. Randomized clinical trials (RCTs) conducted in neurocritical care (NCC) patients have produced controversial results [5,6,7,8,9]. Previous meta-analyses focusing on patients with ABI or traumatic brain injury (TBI) have suggested no significant differences in the occurrence of unfavorable functional outcomes between liberal and restrictive transfusion strategies [4,10,11,12,13]. Recently, a large RCT conducted in patients with subarachnoid hemorrhage (SAH) reported that a liberal transfusion strategy did not significantly reduce the risk of unfavorable neurological outcomes [9].

This meta-analysis aims to synthesize data from RCTs to evaluate the impact of liberal versus restrictive transfusion strategies on neurological functional outcome, mortality, and safety in patients with ABI. Additionally, given the diversity in the etiology and management of ABIs, we conducted subgroup analyses to explore the potential differences between conditions such as TBI and SAH. By providing a comprehensive and updated analysis, we seek to clarify the role of transfusion thresholds in optimizing care for this vulnerable population.

## 2. Materials and Methods

### 2.1. Study Design

This systematic review and meta-analysis was conducted according to the Preferred Reporting Items for Systematic Reviews and Meta-analyses (PRISMA) reporting guidelines in the Supplementary Materials. The protocol was preregistered in the International Prospective Register of Systematic Reviews (PROSPERO) database (CRD42024619125).

### 2.2. Eligibility Criteria

We included all peer-reviewed, English-language RCTs published as full articles. Studies analyzing subgroups derived from previously published RCTs were also included. The study population included adult patients (aged ≥18 years) who were admitted to an intensive care unit (ICU) with an ABI and had hemoglobin values below 10 g/dL. ABIs included intracerebral hemorrhage (ICH), SAH, and TBI.

### 2.3. Intervention

The intervention of interest was a liberal transfusion strategy, with hemoglobin thresholds ranging from ≤9 g/dL to ≤10 g/dL across the included trials. This was compared with a restrictive transfusion strategy, which utilized hemoglobin thresholds ranging from ≤7 g/dL to ≤8 g/dL.

### 2.4. Outcome Measures

The primary outcome of interest was neurological functional outcome, categorized dichotomously as favorable or unfavorable. Due to the variation in standardized scales used across trials, an unfavorable neurological outcome was defined as a Glasgow Outcome Scale (GOS) score of less than 4, an Extended Glasgow Outcome Scale (GOS-E) score of less than 5, or a modified Rankin Scale (mRS) score greater than 3 at the last follow-up. Secondary outcomes included mortality (both in-hospital and assessed at different time points), ICU length of stay (LOS), hospital LOS, quality of life (QOL), and functional outcome at the last follow-up, measured using the EuroQol Five-Dimension Five-Level Instrument (EQ-5D-5L) and the Functional Independence Measure (FIM) score, respectively. Safety analysis compared adverse event (AE) rates between the two groups. Risk ratios (RRs) were used for dichotomous outcomes, and mean differences (MDs) were used for continuous outcomes. Subgroup analyses were performed for disease and models of transfusion threshold. To assess LOS, we extracted the means and standard deviation (SD) for each participant group. If medians and interquartile ranges (IQRs) were reported, these were converted to means and SDs following the methodologies proposed by Luo et al. and Shi et al. [14,15]. If necessary, we approximated data from reported percentages or figures in the included studies.

### 2.5. Data Sources

We searched the following electronic databases from inception to 18 December 2024: Pubmed, Scopus, and the Cochrane Central Register of Controlled Trials. Following the publication of an additional trial, the search strategy was rerun across all databases to ensure the inclusion of the most up-to-date evidence, despite this step not being specified in the original protocol. Previous reviews and included studies were screened for relevant citations. The search strategy is presented in the Methods section in the Supplementary Materials.

### 2.6. Data Collection and Analysis

Studies identified through the search strategy and citation chaining were uploaded to Rayyan and duplicates removed. Two authors (P.M. and M.A.) independently screened abstracts and categorized them as “excluded”, “included”, and “maybe”, with the latter 2 progressing to the next stage of screening. Full texts of the remaining studies were uploaded to Rayyan for the second stage of screening by the 2 reviewers [16]. Discrepancies were resolved through discussion with a third reviewer (E.C.I.). The final included studies were collated for data extraction and analysis. Two reviewers (P.M. and M.A.) extracted data independently from the eligible studies using a data extraction form. We abstracted the following information: study characteristics, participant characteristics, interventions, comparators, and outcomes.

### 2.7. Subgroup Analysis

We conducted a prespecified subgroup analysis for the primary outcome, stratified by the following disease processes: SAH and TBI.

### 2.8. Risk-of-Bias Assessment

Version 2 of the Cochrane risk-of-bias tool for randomized trials (RoB 2) was used for quality assessment of each study, which consists of 5 domains [17]. Two reviewers (P.M. and M.A.) independently assessed the risk of bias for the primary outcome, classifying each domain and overall risk of bias as low, moderate, serious, or critical risk, with discrepancies resolved by discussion between the 2 authors.

### 2.9. Statistical Analysis

We anticipated substantial between-study heterogeneity and therefore utilized a random-effects model to pool effect sizes. For dichotomous data, we calculated a pooled estimate of the RR with a 95% CI using a random-effects model based on the Mantel–Haenszel method. For continuous data, we calculated a pooled estimate of the MD with a 95% CI using the Inverse Variance method. Heterogeneity was assessed using the Cochran’s Q statistic, with a corresponding *p* value, and quantified using the I^2^ statistic (which described the percentage of total variation across the studies due to heterogeneity rather than chance) [18]. See the Methods section in the Supplementary Materials for the full statistical analysis plan. Between-study variance (τ^2^) was estimated using the Paule–Mandel method. Publication bias was evaluated through visual inspection of funnel plots. Statistical significance was set at a *p* value < 0.05 for all analyses. All analyses were conducted in Review Manager [19]. We assessed confidence in the evidence for the main assessed outcomes using the Grading of Recommendations Assessment, Development, and Evaluation framework (GRADE) [20].

### 2.10. Sensitivity Analyses

We conducted the following sensitivity analyses for the primary outcome to evaluate the robustness of our results: (1) we evaluated neurological outcome by converting all reported functional scores across studies to the GOS, with unfavorable neurological outcomes defined as a GOS score of less than 4 (detailed conversion process provided in the Methods in the Supplementary Materials); (2) we evaluated the effectiveness and safety of liberal transfusion strategies across varying transfusion thresholds; (3) to address potential heterogeneity due to different follow-up periods, we analyzed neurological outcomes at the last follow-up visit among studies with uniform follow-up durations.

## 3. Results

**Figure 1:**
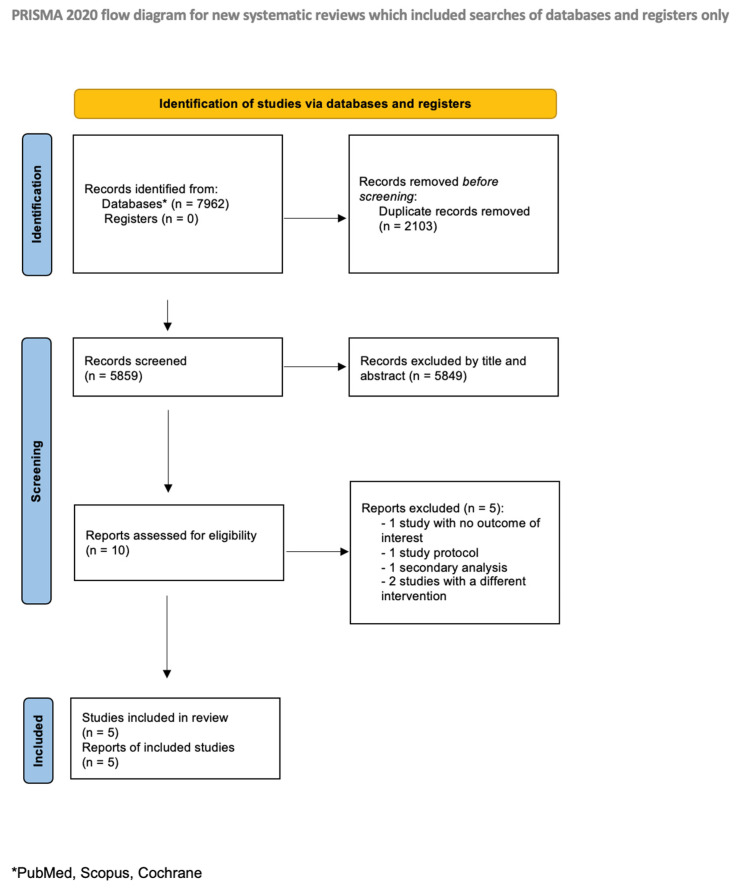
Study selection: PRISMA flowchart presenting the selection of eligible studies.

After 5859 nonduplicated published records were screened, 10 studies fulfilled the inclusion criteria. Five studies were included following full-text assessment for eligibility (Figure 1) [6,7,8,9,21,22]. All five included studies were RCTs, with four included in the analysis of the primary outcome (Table 1).

### 3.1. Primary Outcome

**Figure 2:**
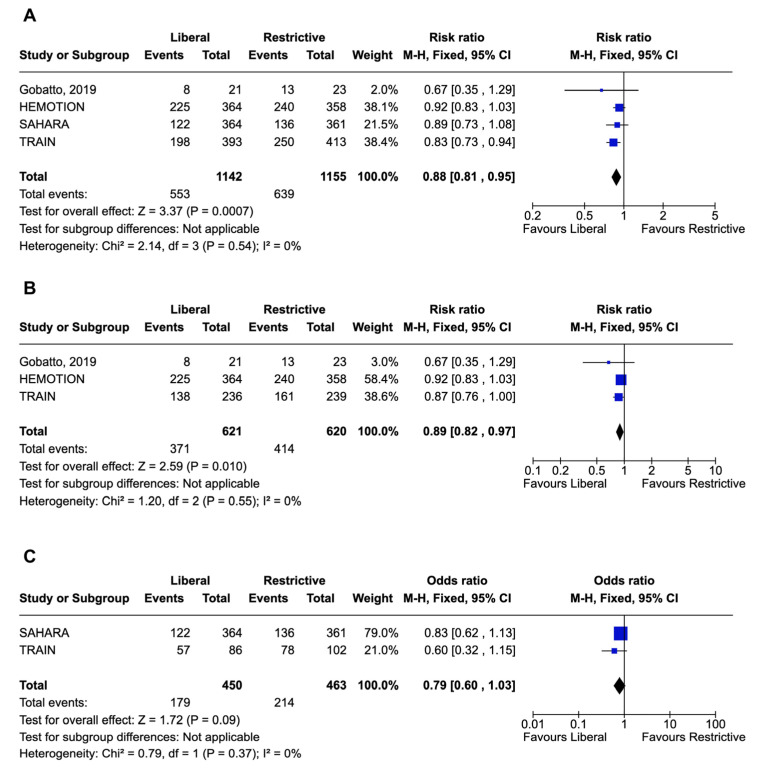
Forest plot of unfavorable neurological outcome and subgroup analyses. Forest plot for the primary outcome in the entire population (**A**), for the subgroup of traumatic brain injury patients (**B**), and for the subgroup of subarachnoid hemorrhage patients (**C**). Squares indicate the point estimates for individual studies, with their size proportional to the statistical weight of each study. Horizontal lines beside the squares indicate the confidence intervals for each study. Diamonds indicate the pooled effect estimates, with their width representing the 95% confidence interval. The vertical line represents the null effect; diamonds that do not cross this line indicate a statistically significant effect [6,7,8,9].

Four studies including 2297 patients were included in the meta-analysis of unfavorable neurological functional outcome. Critically ill adults with ABI receiving liberal transfusion threshold (n = 1142) had a significantly lower risk of unfavorable functional outcome (RR, 0.88; 95% CI, 0.82–0.95; *p* = 0.0009; I^2^ 0%), compared with those receiving restrictive strategy (n = 1155) at the last follow-up (Figure 2A). No heterogeneity was described for the primary outcome (Figure 2A). Three trials had low risk of bias [6,7,9], while one trial had some concerns (Figure S1) [8].

### 3.2. Sensitivity Analyses of the Primary Outcome

Sensitivity analyses concerning the primary outcome are summarized in Figure S2. The heterogeneity of the measures used across trials to assess neurological recovery does not appear to influence the significance of the results. This was confirmed by the pooled analysis of data after conversion to the GOS for all included studies, using a GOS score of less than 4 to define unfavorable functional outcome (RR, 0.88; 95% CI, 0.82–0.95; *p* = 0.0005; I^2^ = 0%) (Figure S2 in the Supplementary Materials).

When evaluating different liberal transfusion threshold strategies, a more conservative approach with a cut-off of ≤9 g/dL demonstrated a significantly lower risk of unfavorable functional outcome compared with the restrictive strategy (RR, 0.88; 95% CI, 0.82–0.95; *p* = 0.0009; I^2^ = 0%). In contrast, a non-significant lower risk was found for a cut-off of ≤10 g/dL (RR, 0.91; 95% CI, 0.83–1.01; *p* = 0.06; I^2^ = 0%) (Figure S2).

Finally, the sensitivity analysis of the three studies with a 6-month follow-up duration confirmed a significantly lower risk of unfavorable functional outcome with the liberal approach compared to the restrictive strategy (RR, 0.87; 95% CI, 0.80–0.95; *p* = 0.001; I^2^ = 7%) (Figure S2).

### 3.3. Subgroup Analyses of the Primary Outcome

The subgroup analysis of TBI demonstrated a significantly reduced risk of unfavorable neurological functional outcome in patients treated with a liberal transfusion strategy compared to a restrictive strategy (RR, 0.89; 95% CI, 0.82–0.97; *p* = 0.010; I^2^ = 0%) (Figure 2B). In contrast, the subgroup analysis of SAH did not show significant differences between groups in terms of the primary outcome (RR, 0.79; 95% CI, 0.60–1.03; *p* = 0.09; I^2^ = 0%) (Figure 2C). A subgroup analysis of ICH patients was not performed due to the small number of patients enrolled in one single RCT.

### 3.4. Secondary Outcomes

**Figure 3:**
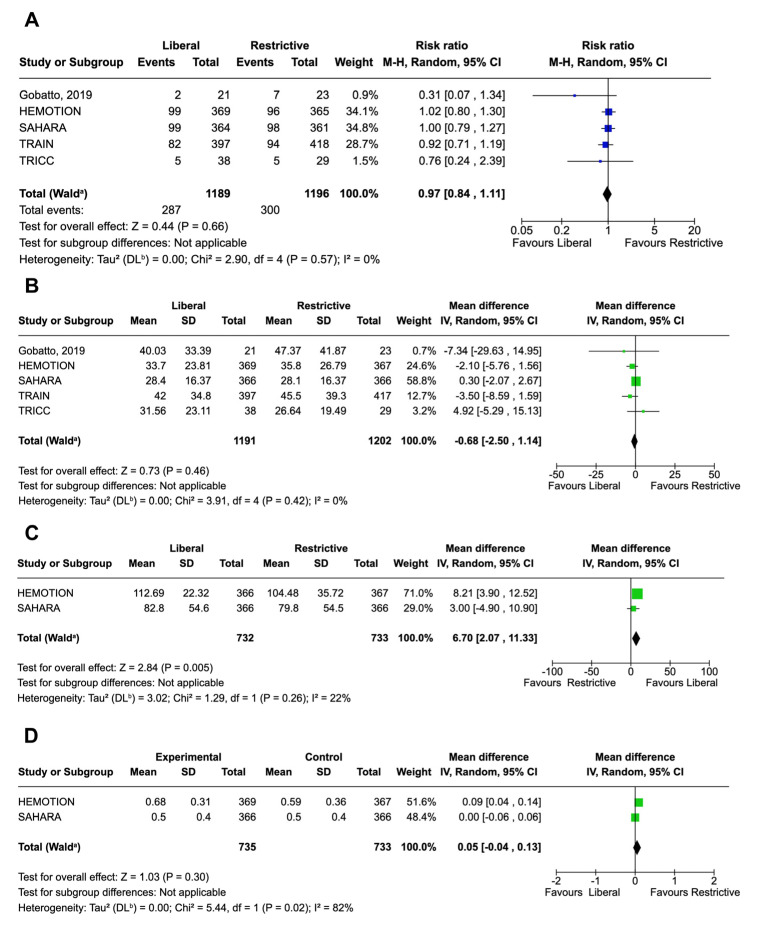
Forest plot for mortality and other secondary outcomes. (**A**) displays the forest plot for mortality at the last follow-up, (**B**) presents the forest plot for hospital length of stay, (**C**) illustrates the forest plot for the Functional Independence Measure, (**D**) shows the forest plot for quality of life. Squares indicate the point estimates for individual studies, with their size proportional to the statistical weight of each study. Horizontal lines beside the squares indicate the confidence intervals for each study. Diamonds indicate the pooled effect estimates, with their width representing the 95% confidence interval. The vertical line represents the null effect; diamonds that do not cross this line indicate a statistically significant effect [6,7,8,9,22].

All included studies reported data on mortality at different follow-up. Mortality rates at the longest follow-up until 12 months were similar between groups (RR, 0.97; 95% CI, 0.84–1.11; *p* = 0.66; I^2^ 0%) (Figure 3A). No differences were observed in in-hospital mortality (RR, 1.02; 95% CI, 0.79–1.31; *p* = 0.89; I^2^ 21%), nor when mortality was assessed at 1 month (RR, 0.75; 95% CI, 0.39–1.43; *p* = 0.38; I^2^ 34%) or 6 months (RR, 0.71; 95% CI, 0.24–2.07; *p* = 0.53; I^2^ 60%) (Figure S3 in the Supplementary Materials). There were no statistically significant differences in the pooled MD for either ICU (MD, −0.30 days; 95% CI, –1.32–0.72; *p* = 0.56; I^2^ 18%) (Figure S3) or hospital LOS (MD, −0.68 days; 95% CI, –2.50–1.14; *p* = 0.46; I^2^ 0%) (Figure 3A,B). Functional independence, expressed as an FIM score, was reported in 2 studies, including a total of 1465 patients. Critically ill adults with ABI who received liberal transfusion strategy had significantly higher scores (MD, 6.70; 95% CI, 2.07–11.33; *p* = 0.005; I^2^ 22%), compared to the control group (Figure 3C), although in the same studies, no differences were noted in the EQ-5D-5L (MD, 0.05; 95% CI, −0.04–0.13; *p* = 0.30; I^2^ 82%) (Figure 3D). It is important to consider, when reviewing these results, that in the conversion from median and IQR to mean and SD, the data were found to be skewed for the outcomes of LOS, functional independence and QOL, indicating a non-normal distribution that may have impacted the accuracy of this approximation.

### 3.5. Safety Analysis

We analyzed several AEs reported in the included trials, showing that sepsis or septic shock occurred less frequently in the liberal group (RR, 0.68; 95% CI, 0.50–0.92; *p* = 0.01). No further significant differences between groups were found in all other analyzed AEs (Figures S4 and S5).

## 4. Discussion

Patients with ABI are particularly vulnerable to cerebral ischemia due to impaired autoregulation and high metabolic demands. As the brain relies almost entirely on oxygen-dependent glucose metabolism, adequate oxygen delivery is critical. Anemia may worsen cerebral hypoxia by reducing hemoglobin-mediated oxygen transport, potentially leading to poorer outcomes. Higher hemoglobin levels and red blood cell transfusion may be associated with higher cerebral oxygen delivery and decreased cerebral ischemic burden [23]. These considerations have supported the rationale for evaluating liberal transfusion thresholds in this population.

This systematic review and meta-analysis of five RCTs demonstrated that a liberal transfusion threshold is associated with improved neurological functional outcomes in critically ill adults with ABIs. Our results contradict earlier evidence from prior meta-analyses in NCC patients [10,11,12,13]. The observed discrepancy can be attributed, in part, to the larger sample size in our analysis and, importantly, to the exclusion of the RCT by Robertson et al. [5], which was included in prior meta-analyses despite notable differences in the intervention studied (i.e., erythropoietin in conjunction with transfusion thresholds).

For the primary outcome, we combined various standardized scales reported across the trials to define unfavorable neurological functional outcome. Among the included studies, two trials used the GOS-E but applied different cut-offs (GOS-E ≤4 or ≤5) to define unfavorable outcome. To standardize the data, we extracted the number of patients for each score and adopted a uniform definition, considering an unfavorable outcome as a GOS-E score ≤4. Furthermore, to mitigate potential heterogeneities arising from the use of different measures, we conducted a sensitivity analysis on the primary outcome by converting each standardized scale to the equivalent GOS score, defining an unfavorable neurological outcome as GOS ≤3.

In consideration of the different transfusion strategies applied across the analyzed studies, reflecting the wide spectrum of clinical scenarios encompassed by our ABI population, a sensitivity analysis evaluating different liberal transfusion thresholds (9 g/dL and 10 g/dL) was conducted, suggesting that a more conservative threshold of 9 g/dL may be more effective. Accordingly, among the included studies, both RCT using the 9 g/dL cut-off for the liberal transfusion strategy reported a significant reduction in the likelihood of unfavorable neurological outcomes compared with the restrictive approach [6,7]. These findings align with prior evidence indicating that higher hemoglobin levels in certain ABI populations may be associated with poorer outcomes [24]

ABIs encompass a diverse range of conditions, and this meta-analysis provides evidence that a liberal transfusion threshold is associated with a significantly reduced risk of unfavorable neurological outcome within the TBI subgroup. Additionally, this is the first meta-analysis to perform a pooled analysis on patients with spontaneous SAH. While a nominally reduced risk of unfavorable neurological outcomes was observed in SAH patients receiving a liberal transfusion strategy compared to a restrictive approach, the difference did not reach statistical significance. However, this analysis was possibly underpowered, as it included only two RCTs analyzing patients with SAH, with a pooled sample size smaller than that of the TBI subgroup [7,9]. This suggests that further RCTs are warranted to evaluate the effectiveness of a liberal transfusion strategy in this etiological group. Finally, no subgroup analysis was conducted on the ICH group, as only one trial included these patients [7].

This meta-analysis found no significant difference in mortality, both assessed in-hospital and at different timepoints. This suggests that a liberal transfusion strategy may promote better neurological recovery among survivors without affecting overall survival. However, while the evidence for the primary outcome was rated as moderate, the evidence for mortality was rated as low according to GRADE.

In our analysis, we observed a significantly higher pooled functional independence score in the liberal transfusion group. However, this improvement in functional independence did not translate into significant differences in QOL. This discrepancy may be attributed to several factors not directly addressed in this meta-analysis but highlighted in previous research. These include the influence of ABI type and severity on QOL recovery [25], as well as socioeconomic variables such as insurance status and access to rehabilitation services [26,27]. Moreover, the “disability paradox” underscores a disconnect between functional disability and reported well-being, with some patients experiencing significant disabilities yet reporting unexpectedly high QOL, challenging the assumption that functional limitations invariably correlate with diminished QOL [28].

Importantly, this study extensively reviewed the safety and incidence of adverse events between groups. This meta-analysis highlights that sepsis and septic shock were less frequent in the liberal strategy group, although the observed relationship between anemia and the risk of developing sepsis/septic shock remains undefined. Previous research has indicated that anemia is common in septic patients and is often associated with worse prognostic outcomes. For instance, lower hemoglobin levels at admission have been linked to higher mortality rates in sepsis patients, suggesting that anemia may exacerbate the severity of sepsis rather than directly contributing to its onset [29,30,31,32]. Therefore, while current evidence does not support a direct causal link between anemia and the development of sepsis or septic shock, its presence in septic patients is widely regarded as a marker of disease severity. Given this context, the higher severity of sepsis associated with lower hemoglobin levels may influence the likelihood of a sepsis or septic shock diagnosis, potentially leading to the observed statistically significant difference in sepsis/septic shock incidence between the two groups.

Conversely, no differences were observed in the remaining safety outcomes, with no increase in the incidence of thromboembolic events or stroke between groups. Previous studies have yielded contrasting results: one RCT reported an increase in venous thromboembolism in the liberal strategy group [5], while another RCT highlighted a reduced incidence of ischemic stroke in the same group [7]. Unfortunately, in the present meta-analysis, a detailed distinction between ischemic and hemorrhagic stroke was not possible due to the inconsistent definitions of this adverse outcome in some of the included studies [6,8].

Potential limitations of this study include the variability in definitions of the primary outcome, as neurological outcomes were assessed using three different standardized measures. However, sensitivity analysis converting different scales to the GOS yielded consistent results. The heterogeneity within our ABI population, with most studies focusing on TBI patients and the underrepresentation of ICH patients, may limit the generalizability of our findings to all ABI patients. Additionally, differences in the intervention, with slightly varied transfusion strategies employed in the liberal and restrictive groups, should be taken into account. Finally, slight variations in follow-up durations across included studies should be also considered, although a sensitivity analysis including studies with uniform follow-up durations confirmed our findings.

## 5. Conclusions

This systematic review and meta-analysis found that a liberal transfusion threshold in patients with ABI and anemia is associated with improved favorable neurological functional outcomes compared to a restrictive approach, with no safety issues. Notably, this benefit was evident in the subgroup of patients with TBI. However, additional research is required to determine whether this strategy can provide similar advantages in other subgroups of NCC patients and to identify the optimal cut-off for a liberal transfusion threshold to maximize clinical benefits.

## Figures and Tables

**Table 1:** Baseline characteristics of included studies.

Study	Restrictive(R) Threshold	Liberal (L) Threshold	Unfavorable Neurological Outcome (Definition)	FU, mo	Patients n°R/L	Male, % R/L	Age ^†^, y R/L	GCS ^†^ at Admission R/L	Injury Severity Score at Admission ^†^ R/L	Hb ^†^ at Admission R/L	Time Injury to Randomization	Patients Transfused Before Randomization R/L	Surgical or Interventional Radiological Procedure R/L
Gobatto 2019 [6]	<7 g/dL	<9 g/dL	GOS ≤3	6	23/21	87/95	36/33	5/4	31/28	12.5/12	65/75 §	15/9	19/21
TRAIN 2024 [7]	≤7 g/dL	≤9 g/dL	GOS-E ≤5	6	423/397	53.4/54.9	51/52	6/7	NA	11.9/11.8	NA	NA	135/110
HEMOTION 2024 [8]	≤7 g/dL	≤10 g/dL	GOS-E ≤4	6	367/369	69.5/75.9	48.4/48.9	4/4	32/30	13.1/13.3	56/55 §	67/57	226/255
TRICC 1999 [22]	<7 g/dL	<10 g/dL	NA	2	29/38	90/74	41.7/39.8	7.3/7.5	29.8/31.3	NA	NA	NA	NA
SAHARA 2024 [9]	≤8 g/dL	≤10 g/dL	mRS ≥4	12	366/366	18.3/18.3	59.5/59.3	NA	NA	9.3/9.4	3.8/3.6 ‡	28/40	NA

Abbreviations: FU = follow-up; GOS = Glasgow Outcome Scale; GOS-E = Glasgow Outcome Scale–Extended; Hb = hemoglobin; HF = heart failure; ICH = intracranial cerebral hemorrhage; GCS = Glasgow coma scale; Mo = months. Note: † mean; ‡ days; § hours; GCS-motor.

## Data Availability

The data supporting this meta-analysis are derived from previously published studies and publicly available datasets. All included studies are cited within the manuscript. The extracted data and analysis scripts are available upon reasonable request from the corresponding author.

## Supplementary Materials

The following supporting information can be downloaded at: https://www.mdpi.com/article/10.3390/jcm14103487/s1. Figure S1. Risk of bias assessment; Figure S2. Sensitivity analyses of the primary outcome; Figure S3. Forest plot of in-hospital mortality, length of stay in intensive care unit and 1 and 6 months mortality; Figure S4. Forest plot of adverse events; Figure S5. Forest plot of adverse events; Figure S6. Funnel plot of the primary outcome; Figure S7. Grading of Recommendations Assessment, Development and Evaluation framework (GRADE) for the primary outcome and mortality. PRISMA 2020 Checklist. References [33,34] are cited in the Supplementary Materials.

## Abbreviations

The following abbreviations are used in this manuscript:

ABI	acute brain injury
EQ-5D-5L	EuroQol Five-Dimension, Five-Level Instrument
FIM	functional independence measure
GOS	Glasgow Outcome Scale
GOS-E	Glasgow Outcome Scale-Extended
GRADE	Grading of Recommendations Assessment, Development and Evaluation framework
ICH	intracerebral hemorrhage
ICU	intensive care unit
IQR	interquartile range
LOS	length of stay
MD	mean difference
NCC	neurocritical care
QOL	quality of life
RCT	randomized control trial
ROB	risk of bias
SAH	subarachnoid hemorrhage
SD	standard deviation
TBI	traumatic brain injury

## References

[1] Lelubre C., Bouzat P., Crippa I.A., Taccone F.S. (2016). Anemia management after acute brain injury. Crit. Care.

[2] Terrett L.A., McIntyre L., Turgeon A.F., English S.W. (2023). Anemia and Red Blood Cell Transfusion in Aneurysmal Subarachnoid Hemorrhage. Neurocrit. Care.

[3] Sutton D.H., Raines D.A. (2017). The Risks Associated with Red Blood Cell Transfusion: Implications for Critical Care Practice. Crit. Care Nurs. Clin. N. Am..

[4] Montgomery E.Y., Barrie U., Kenfack Y.J., Edukugho D., Caruso J.P., Rail B., Hicks W.H., Oduguwa E., Pernik M.N., Tao J. (2022). Transfusion Guidelines in Traumatic Brain Injury: A Systematic Review and Meta-Analysis of the Currently Available Evidence. Neurotrauma Rep..

[5] Robertson C.S., Hannay H.J., Yamal J.-M., Gopinath S., Goodman J.C., Tilley B.C., Baldwin A., Lara L.R., Saucedo-Crespo H., the Epo Severe TBI Trial Investigators (2014). Effect of Erythropoietin and Transfusion Threshold on Neurological Recovery After Traumatic Brain Injury: A randomized clinical trial. JAMA.

[6] Gobatto A.L.N., Link M.A., Solla D.J., Bassi E., Tierno P.F., Paiva W., Taccone F.S., Malbouisson L.M. (2019). Transfusion requirements after head trauma: A randomized feasibility controlled trial. Crit. Care.

[7] Taccone F.S., Rynkowski C.B., Møller K., Lormans P., Quintana-Díaz M., Caricato A., Ferreira M.A.C., Badenes R., Kurtz P., Søndergaard C.B. (2024). Restrictive vs Liberal Transfusion Strategy in Patients with Acute Brain Injury: The TRAIN Randomized Clinical Trial. JAMA.

[8] Turgeon A.F., Fergusson D.A., Clayton L., Patton M.-P., Neveu X., Walsh T.S., Docherty A., Malbouisson L.M., Pili-Floury S., English S.W. (2024). Liberal or Restrictive Transfusion Strategy in Patients with Traumatic Brain Injury. N. Engl. J. Med..

[9] English S.W., Delaney A., Fergusson D.A., Chassé M., Turgeon A.F., Lauzier F., Tuttle A., Sadan O., Griesdale D.E., Redekop G. (2024). Liberal or Restrictive Transfusion Strategy in Aneurysmal Subarachnoid Hemorrhage. N. Engl. J. Med..

[10] Boutin A., Chassé M., Shemilt M., Lauzier F., Moore L., Zarychanski R., Griesdale D., Desjardins P., Lacroix J., Fergusson D. (2016). Red Blood Cell Transfusion in Patients with Traumatic Brain Injury: A Systematic Review and Meta-Analysis. Transfus. Med. Rev..

[11] Florez-Perdomo W.A., García-Ballestas E., Martinez-Perez R., Agrawal A., Deora H., Joaquim A.F., Quiñones-Ossa G.A., Moscote-Salazar L.R. (2021). Hemoglobin levels as a transfusion criterion in moderate to severe traumatic brain injury: A systematic review and meta-analysis. Br. J. Neurosurg..

[12] Yu Y., Fu Y., Li W., Sun T., Cheng C., Chong Y., Han R., Cui W. (2024). Red blood cell transfusion in neurocritical patients: A systematic review and meta-analysis. BMC Anesthesiol..

[13] Yuan X., Zhang S., Wan J., Chen C., Wang P., Fan S., Liu Y., Yang J., Hou J., You Q. (2024). Efficacy of restrictive versus liberal transfusion strategies in patients with traumatic brain injury: A systematic review and meta-analysis of randomized controlled trials. Ann. Intensiv. Care.

[14] Luo D., Wan X., Liu J., Tong T. (2018). Optimally estimating the sample mean from the sample size, median, mid-range, and/or mid-quartile range. Stat. Methods Med. Res..

[15] Shi J., Luo D., Weng H., Zeng X., Lin L., Chu H., Tong T. (2020). Optimally estimating the sample standard deviation from the five-number summary. Res. Synth. Methods.

[16] Ouzzani M., Hammady H., Fedorowicz Z., Elmagarmid A. (2016). Rayyan—A web and mobile app for systematic reviews. Syst. Rev..

[17] Sterne J.A.C., Savović J., Page M.J., Elbers R.G., Blencowe N.S., Boutron I., Cates C.J., Cheng H.Y., Corbett M.S., Eldridge S.M. (2019). RoB 2: A revised tool for assessing risk of bias in randomised trials. BMJ.

[18] Moro P., Lattanzi S., Beier C.P., Di Bonaventura C., Irelli E.C. (2024). Cognitive behavioral therapy in adults with functional seizures: A systematic review and meta-analysis of randomized controlled trials. Epilepsy Behav..

[19] (2024). Review Manager (RevMan) [Computer Program].

[20] Guyatt G.H., Oxman A.D., Vist G.E., Kunz R., Falck-Ytter Y., Alonso-Coello P., Schünemann H.J. (2008). GRADE: An emerging consensus on rating quality of evidence and strength of recommendations. BMJ.

[21] McIntyre L.A., Fergusson D.A., Hutchison J.S., Pagliarello G., Marshall J.C., Yetisir E., Hare G.M.T., Hébert P.C., Canadian Critical Care Trials Group (2006). Effect of a Liberal Versus Restrictive Transfusion Strategy on Mortality in Patients with Moderate to Severe Head Injury. Neurocrit. Care.

[22] Hébert P.C., Wells G., Blajchman M.A., Marshall J., Martin C., Pagliarello G., Tweeddale M., Schweitzer I., Yetisir E. (1999). A multicenter, randomized, controlled clinical trial of transfusion requirements in critical care. Transfusion Requirements in Critical Care Investigators, Canadian Critical Care Trials Group. N. Engl. J. Med..

[23] English S.W., McIntyre L. (2018). Is hemoglobin good for cerebral oxygenation and clinical outcome in acute brain injury?. Curr. Opin. Crit. Care.

[24] Prisco L., Iscra F., Ganau M., Berlot G. (2012). Early predictive factors on mortality in head injured patients: A retrospective analysis of 112 traumatic brain injured patients. J. Neurosurg. Sci..

[25] Mamman R., Grewal J., Garrone J.N., Schmidt J. (2024). Biopsychosocial factors of quality of life in individuals with moderate to severe traumatic brain injury: A scoping review. Qual. Life Res..

[26] Sonesson B., Kronvall E., Säveland H., Brandt L., Nilsson O.G. (2018). Long-term reintegration and quality of life in patients with subarachnoid hemorrhage and a good neurological outcome: Findings after more than 20 years. J. Neurosurg..

[27] Patel M.B., Wilson L.D., Bregman J.A., Leath T.C., Humble S.S., Davidson M.A., de Riesthal M.R., Guillamondegui O.D. (2015). Neurologic Functional and Quality of Life Outcomes after TBI: Clinic Attendees versus Non-Attendees. J. Neurotrauma.

[28] Helmrich I.R.A.R., van Klaveren D., Andelic N., Lingsma H., Maas A., Menon D., Polinder S., Røe C., Steyerberg E.W., Van Veen E. (2022). Discrepancy between disability and reported well-being after traumatic brain injury. J. Neurol. Neurosurg. Psychiatry.

[29] Zhu J., Dong Y., Liao P., Yin X., He J., Guo L. (2023). Prognostic value of hemoglobin in patients with sepsis: A systematic review and meta-analysis. Heart Lung.

[30] Peng H., Su Y., Luo J., Ding N. (2024). Association between admission hemoglobin level and prognosis in sepsis patients based on a critical care database. Sci. Rep..

[31] Qi D., Peng M. (2021). Early Hemoglobin Status as a Predictor of Long-Term Mortality for Sepsis Patients in Intensive Care Units. Shock.

[32] Kristof K., Büttner B., Grimm A., Mewes C., Schmack B., Popov A.F., Ghadimi M., Beissbarth T., Hinz J., Bergmann I. (2018). Anaemia requiring red blood cell transfusion is associated with unfavourable 90-day survival in surgical patients with sepsis. BMC Res. Notes.

[33] Gaastra B., Ren D., Alexander S., Awad I.A., Blackburn S., Doré S., Hanley D., Nyquist P., Bulters D., Galea I. (2022). Evidence-based interconversion of the Glasgow Outcome a.nd modified Rankin scales: Pitfalls and best practices. J. Stroke Cerebrovasc. Dis..

[34] Page M.J., McKenzie J.E., Bossuyt P.M., Boutron I., Hoffmann T.C., Mulrow C.D., Shamseer L., Tetzlaff J.M., Akl E.A., Brennan S.E. (2021). The PRISMA 2020 statement: An updated guideline for reporting systematic reviews. BMJ.

