# Supplementary material for: Transfusion Thresholds and Neurological Functional Outcome After Acute Brain Injury: An Updated Systematic Review and Meta-Analysis of Randomized Clinical Trials
Source: J Clin Med. 2025 May 16;14(10):3487. doi: 10.3390/jcm14103487 (PMC12112058; doi:10.3390/jcm14103487)
Supplement: Supplementary file 1 [file jcm-14-03487-s001.zip › jcm-3527015-supplementary.pdf]

Supplement Material

Transfusion Thresholds and Neurological Functional Outcome After Acute Brain Injury: An Updated Systematic Review and Meta-Analysis of Randomized Clinical Trials

Methods:

Data Sources

We searched the following electronic databases from inception to December 18, 2024: Pubmed, Scopus and the Cochrane Central Register of Controlled Trials. Following the publication of an additional trial, the search strategy was rerun across all databases to ensure the inclusion of the most up-to-date evidence, despite this step not being specified in the original protocol. Previous reviews and included studies were screened for relevant citations. The search strategy was the following: ("transfusion" OR "transfused") AND ("brain injury" OR tbi OR "head injury" OR "head trauma" OR "cerebral injury" OR "intracranial injury" OR "subarachnoid hemorrhage" OR sah OR "intracranial hemorrhage" OR "intracerebral hemorrhage" OR ich).

Statistical analysis

We anticipated substantial between-study heterogeneity and therefore utilized a random-effects model to pool effect sizes. For dichotomous data, we calculated a pooled estimate of the RR with a 95% CI using a random-effects model based on the Mantel-Haenszel method. For continuous data, we calculated a pooled estimate of the MD with a 95% CI using the Inverse Variance method. The MD was used because LOS, QOL, and functional independence were consistently measured across studies using the same units (e.g., days) or standardized assessment tools, such as the EQ-5D-5L and the FIM score, respectively. Heterogeneity was assessed using the Cochran's Q statistic, with a corresponding *P* value, and quantified using the I2 statistic (which described the percentage of total variation across the studies due to heterogeneity rather than chance). *I*2 was calculated from the basic results obtained from a typical meta-analysis as $I2 = 100\% \times (Q - df) / Q$, where Q is the Cochran heterogeneity statistic. A value of 0% indicates no observed heterogeneity, and larger values indicate increasing heterogeneity: low (I2 = 1–25%), moderate (I2 = 26–50%), or high (I2 > 50%). Between-study variance (τ2) was estimated using the Paule-Mandel method. Subgroup analyses were performed using fixed-effects models, assuming an independent τ2 for each subgroup. Publication bias was evaluated through visual inspection of funnel plots. Statistical significance was set at a P value < 0.05 for all analyses. All analyses were conducted in Review Manager.18 We assessed confidence in the evidence for the main assessed outcomes using the Grading of Recommendations Assessment, Development and Evaluation framework (GRADE).19

Sensitivity analysis

We conducted the following sensitivity analyses for the primary outcome to evaluate the robustness of our results. First, we evaluated neurological outcome by converting all reported functional scores across studies to the GOS (Glasgow outcome scale), with unfavorable neurological outcomes defined as a GOS score of less than 4. Specifically, a Glasgow Outcome Scale extended (GOS-E) score of 7 or 8 score was converted to GOS 5, a GOS-E score of 5 or 6 to GOS 4, and lower GOS-E scores were converted to GOS less than 4. For the modified Rankin Scale (mRS), scores of 0–2

were converted to GOS scores of 4–5, as prior research has shown high concordance between these scoring systems when transitioning from mRS to GOS for these scores.e1 As further sensitivity analysis, a stratified analysis of studies that used different transfusion cut-offs was performed to assess the effectiveness and safety of liberal transfusion strategies across varying transfusion thresholds. Finally, to address potential heterogeneity due to varying follow-up periods, we analyzed neurological outcomes at the last follow-up visit among studies with uniform follow-up durations.

Figure S1. Risk of bias assessment.

Study	Bias from randomization process	Bias due to deviations from intended interventions	Bias due to missing outcome data	Bias in measurement of the outcomes	Bias in selection of the reported result	Overall risk of bias
HEMOTION, 2024	Low	Some concerns	Low	Low	Low	Some concerns
TRAIN, 2024	Low	Low	Low	Low	Low	Low
SAHARA, 2024	Low	Low	Low	Low	Low	Low
Gobatto, 2019	Low	Low	Low	Low	Low	Low

Figure S2. Sensitivity analyses of the primary outcome.

Forest plot of the primary outcome categorized as Glasgow outcome scale≤3, forest plot of the sensitivity analysis with liberal threshold cut-off of ≤9 g/dL (Panel B), forest plot of the sensitivity analysis for the primary outcome with liberal threshold cut-off of ≤10 g/dL (Panel C)

A

Study or Subgroup	Liberal Events	Liberal Total	Restrictive Events	Restrictive Total	Weight	Risk ratio M-H, Random, 95% CI
Gobatto, 2019	8	21	13	23	1.2%	0.67 [0.35 , 1.29]
HEMOTION	225	364	240	358	42.1%	0.92 [0.83 , 1.03]
SAHARA	178	364	197	361	25.0%	0.90 [0.78 , 1.03]
TRAIN	198	393	250	413	31.7%	0.83 [0.73 , 0.94]
**Total**		**1142**		**1155**	**100.0%**	**0.88 [0.82 , 0.95]**
Total events:	609		700			

Test for overall effect: Z = 3.46 (P = 0.0005)
Test for subgroup differences: Not applicable
Heterogeneity: Tau² = 0.00; Chi² = 2.18, df = 3 (P = 0.54); I² = 0%

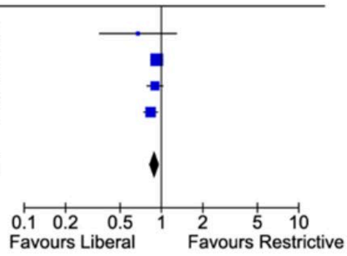

B

Study or Subgroup	Liberal Events	Liberal Total	Restrictive Events	Restrictive Total	Weight	Risk ratio M-H, Random, 95% CI
Gobatto, 2019	8	21	13	23	3.6%	0.67 [0.35 , 1.29]
TRAIN	198	393	250	413	96.4%	0.83 [0.73 , 0.94]
**Total**		**414**		**436**	**100.0%**	**0.83 [0.73 , 0.93]**
Total events:	206		263			

Test for overall effect: Z = 3.04 (P = 0.002)
Test for subgroup differences: Not applicable
Heterogeneity: Tau² = 0.00; Chi² = 0.39, df = 1 (P = 0.53); I² = 0%

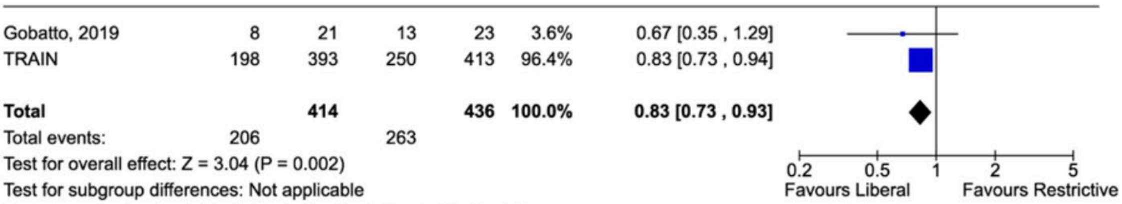

C

Study or Subgroup	Liberal Events	Liberal Total	Restrictive Events	Restrictive Total	Weight	Risk ratio M-H, Random, 95% CI
HEMOTION	225	364	240	358	76.6%	0.92 [0.83 , 1.03]
SAHARA	122	364	136	361	23.4%	0.89 [0.73 , 1.08]
**Total**		**728**		**719**	**100.0%**	**0.91 [0.83 , 1.01]**
Total events:	347		376			

Test for overall effect: Z = 1.85 (P = 0.06)
Test for subgroup differences: Not applicable
Heterogeneity: Tau² = 0.00; Chi² = 0.11, df = 1 (P = 0.75); I² = 0%

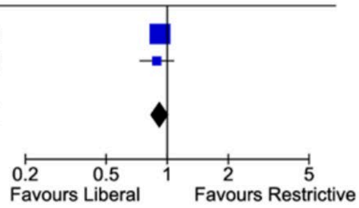

D

Study or Subgroup	Liberal Events	Liberal Total	Restrictive Events	Restrictive Total	Weight	Risk ratio M-H, Fixed, 95% CI
Gobatto, 2019	8	21	13	23	2.5%	0.67 [0.35 , 1.29]
HEMOTION	225	364	240	358	48.6%	0.92 [0.83 , 1.03]
TRAIN	198	393	250	413	48.9%	0.83 [0.73 , 0.94]
**Total**		**778**		**794**	**100.0%**	**0.87 [0.80 , 0.95]**
Total events:	431		503			

Test for overall effect: Z = 3.27 (P = 0.001)
Test for subgroup differences: Not applicable
Heterogeneity: Chi² = 2.14, df = 2 (P = 0.34); I² = 7%

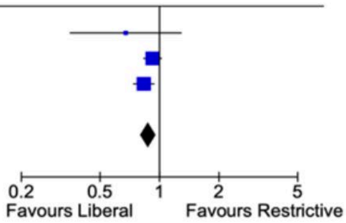

Figure S3. Forest plot of in-hospital mortality, length of stay in intensive care unit and 1 and 6 months mortality.

Forest plot of in-hospital mortality (panel A), forest plot of 1 month mortality (Panel B), forest plot of 6 months mortality (Panel C), forest plot of length of stay in intensive care unit (Panel D)

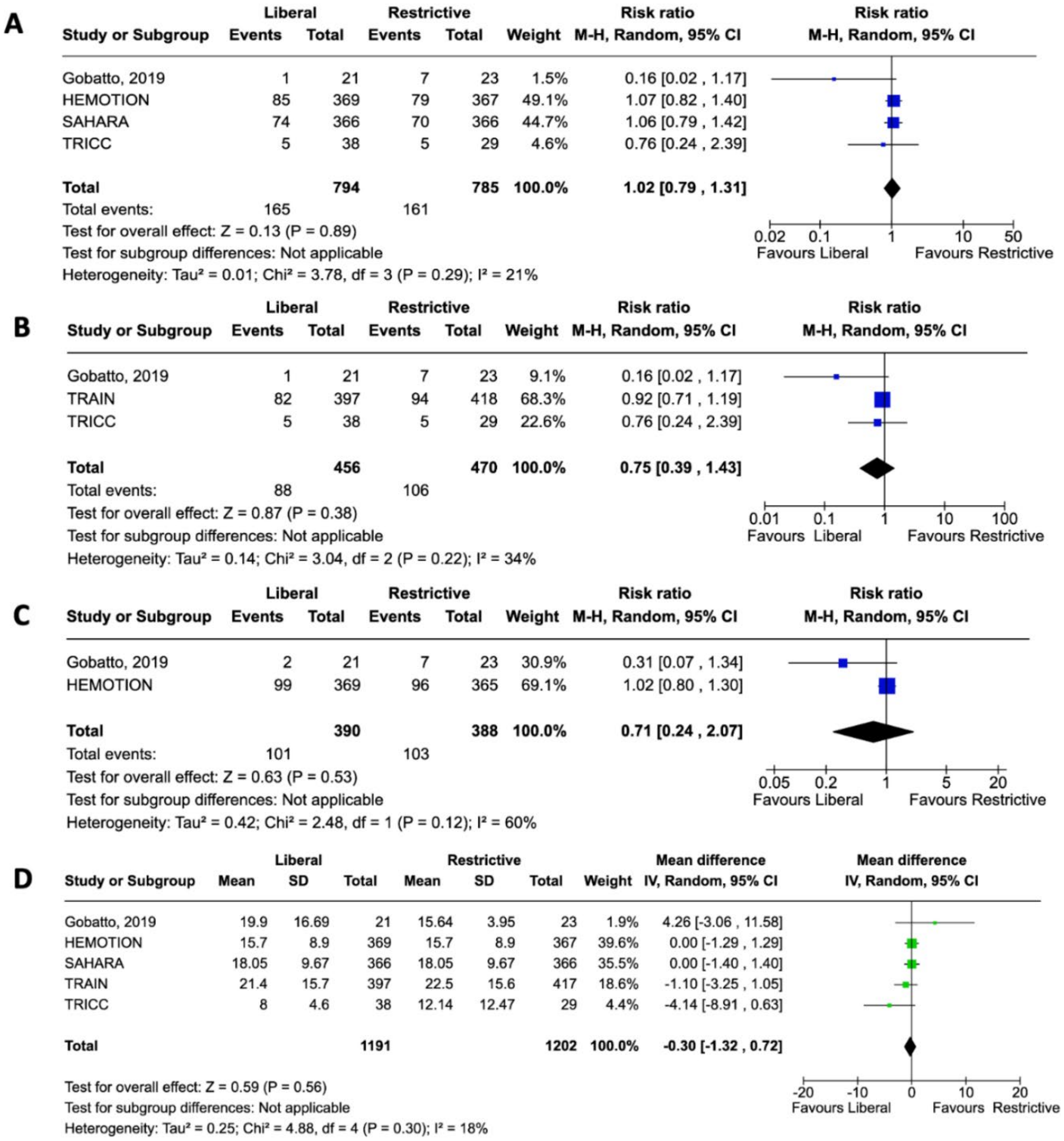

Figure S4. Forest plot of adverse events.

Forest plot of the safety analysis for the adverse outcomes of pneumonia (Panel A), pulmonary embolism (Panel B), venous thromboembolism (Panel C), transfusion reactions (Panel D) and seizures (Panel E)

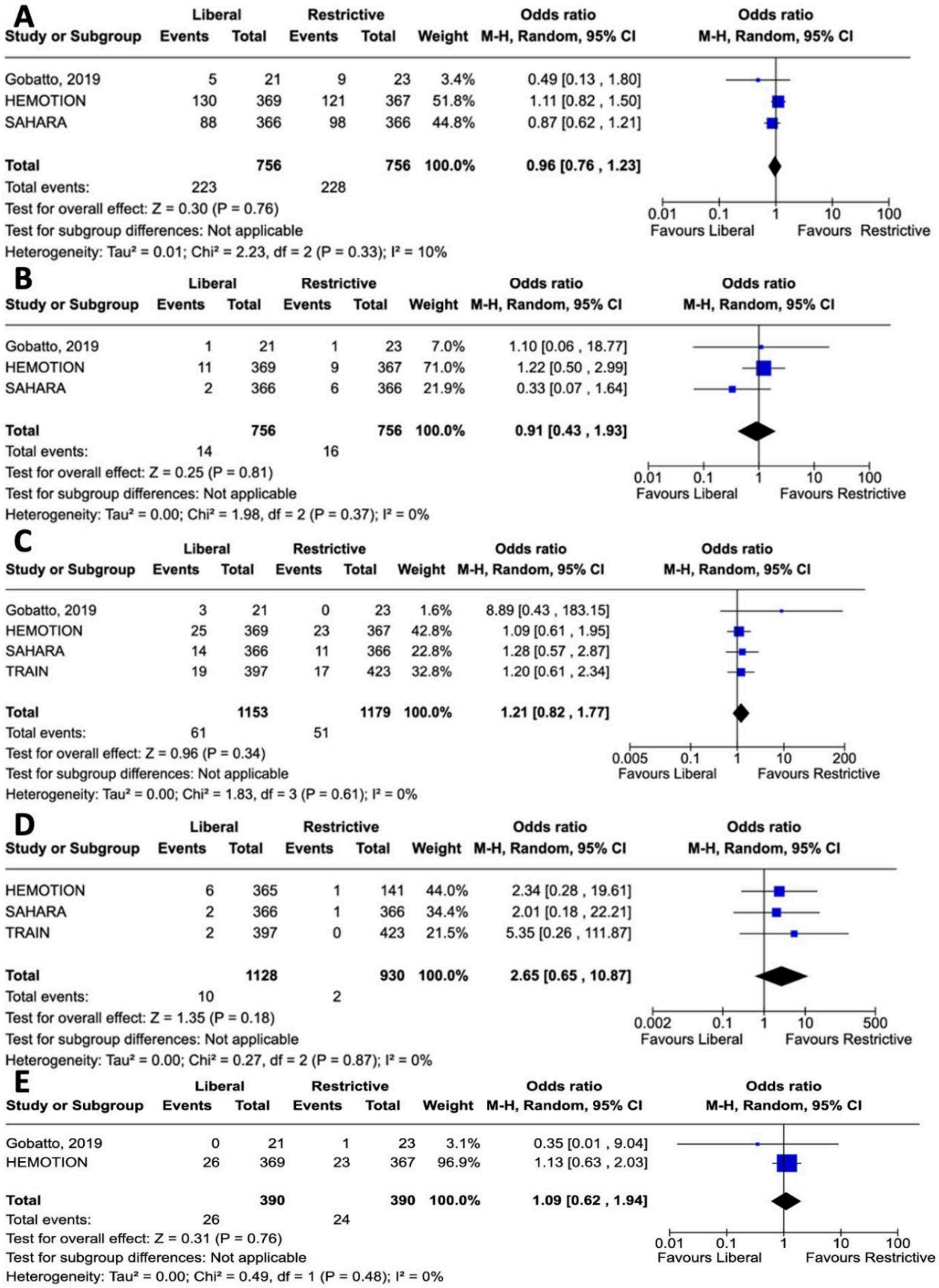

Figure S5. Forest plot of adverse events

Forest plot of the safety analysis for the adverse outcomes of acute respiratory distress syndrome/respiratory failure (Panel A), sepsis/septic shock (Panel B), acute myocardial infarction (Panel C), ischemic/hemorrhagic stroke (Panel D)

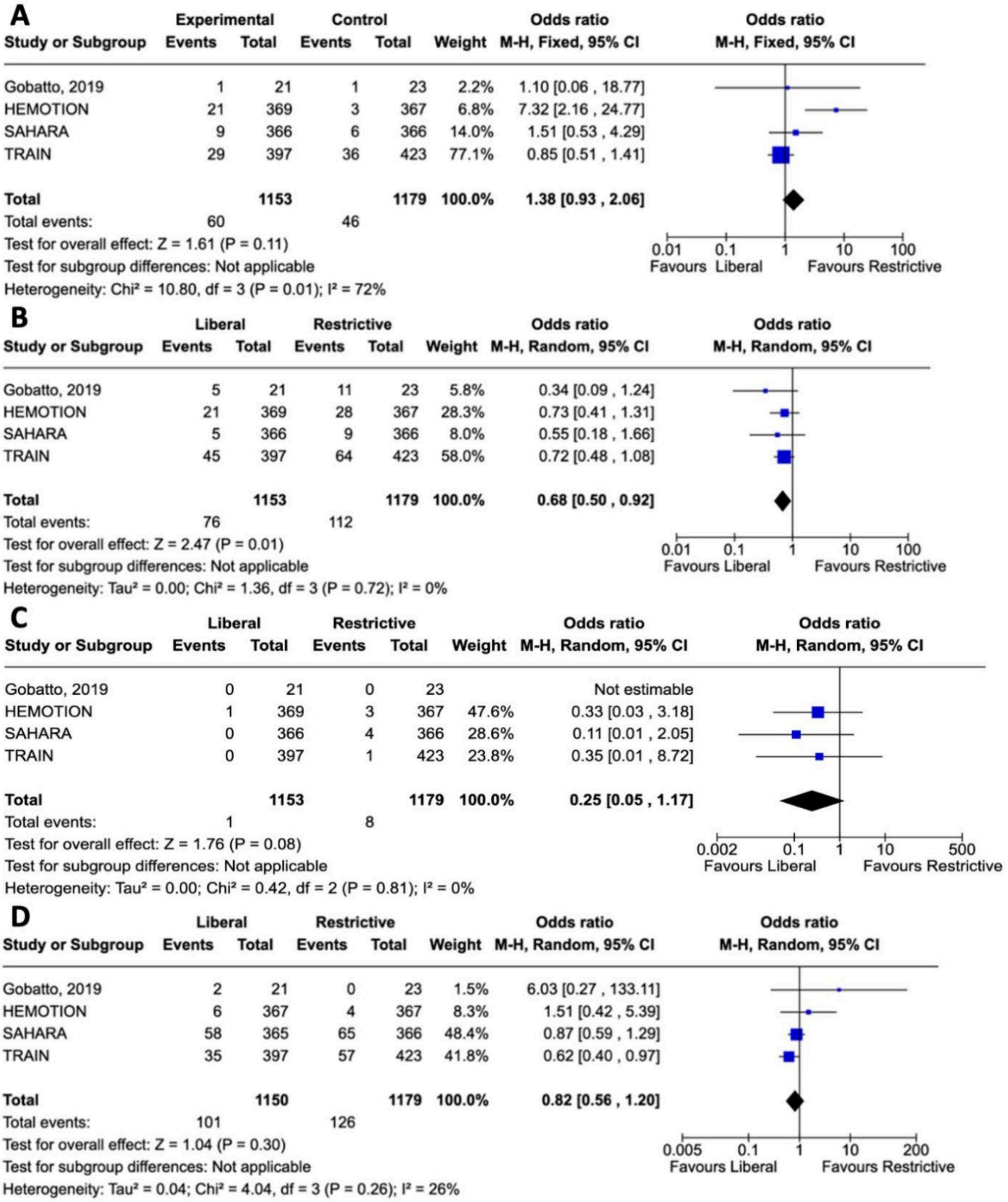

A

Study or Subgroup	Experimental Events	Experimental Total	Control Events	Control Total	Weight	Odds ratio M-H, Fixed, 95% CI
Gobatto, 2019	1	21	1	23	2.2%	1.10 [0.06 , 18.77]
HEMOTION	21	369	3	367	6.8%	7.32 [2.16 , 24.77]
SAHARA	9	366	6	366	14.0%	1.51 [0.53 , 4.29]
TRAIN	29	397	36	423	77.1%	0.85 [0.51 , 1.41]
**Total**		**1153**		**1179**	**100.0%**	**1.38 [0.93 , 2.06]**
Total events:	60		46			

Test for overall effect: Z = 1.61 (P = 0.11)
Test for subgroup differences: Not applicable
Heterogeneity: Chi² = 10.80, df = 3 (P = 0.01); I² = 72%

B

Study or Subgroup	Liberal Events	Liberal Total	Restrictive Events	Restrictive Total	Weight	Odds ratio M-H, Random, 95% CI
Gobatto, 2019	5	21	11	23	5.8%	0.34 [0.09 , 1.24]
HEMOTION	21	369	28	367	28.3%	0.73 [0.41 , 1.31]
SAHARA	5	366	9	366	8.0%	0.55 [0.18 , 1.66]
TRAIN	45	397	64	423	58.0%	0.72 [0.48 , 1.08]
**Total**		**1153**		**1179**	**100.0%**	**0.68 [0.50 , 0.92]**
Total events:	76		112			

Test for overall effect: Z = 2.47 (P = 0.01)
Test for subgroup differences: Not applicable
Heterogeneity: Tau² = 0.00; Chi² = 1.36, df = 3 (P = 0.72); I² = 0%

C

Study or Subgroup	Liberal Events	Liberal Total	Restrictive Events	Restrictive Total	Weight	Odds ratio M-H, Random, 95% CI
Gobatto, 2019	0	21	0	23		Not estimable
HEMOTION	1	369	3	367	47.6%	0.33 [0.03 , 3.18]
SAHARA	0	366	4	366	28.6%	0.11 [0.01 , 2.05]
TRAIN	0	397	1	423	23.8%	0.35 [0.01 , 8.72]
**Total**		**1153**		**1179**	**100.0%**	**0.25 [0.05 , 1.17]**
Total events:	1		8			

Test for overall effect: Z = 1.76 (P = 0.08)
Test for subgroup differences: Not applicable
Heterogeneity: Tau² = 0.00; Chi² = 0.42, df = 2 (P = 0.81); I² = 0%

D

Study or Subgroup	Liberal Events	Liberal Total	Restrictive Events	Restrictive Total	Weight	Odds ratio M-H, Random, 95% CI
Gobatto, 2019	2	21	0	23	1.5%	6.03 [0.27 , 133.11]
HEMOTION	6	367	4	367	8.3%	1.51 [0.42 , 5.39]
SAHARA	58	365	65	366	48.4%	0.87 [0.59 , 1.29]
TRAIN	35	397	57	423	41.8%	0.62 [0.40 , 0.97]
**Total**		**1150**		**1179**	**100.0%**	**0.82 [0.56 , 1.20]**
Total events:	101		126			

Test for overall effect: Z = 1.04 (P = 0.30)
Test for subgroup differences: Not applicable
Heterogeneity: Tau² = 0.04; Chi² = 4.04, df = 3 (P = 0.26); I² = 26%

Figure S6. Funnel plot of the primary outcome

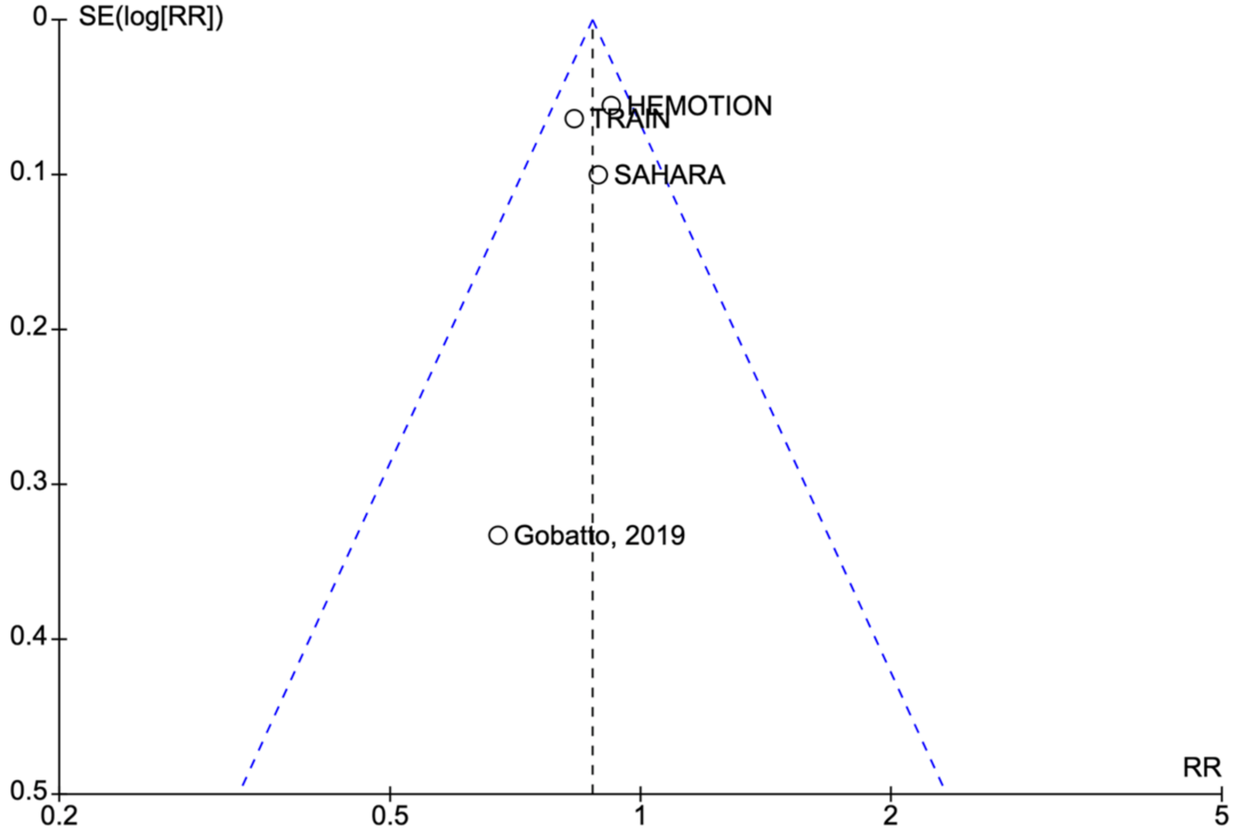

Figure S7. Grading of Recommendations Assessment, Development and Evaluation framework (GRADE) for the primary outcome and mortality

Liberal transfusion strategy compared to restrictive transfusion strategy for acute brain injury

Patient or population: acute brain injury
Setting: Randomized controlled trials in hospital-based settings
Intervention: liberal transfusion strategy
Comparison: restrictive transfusion strategy

Outcomes	№ of participants (studies) Follow-up	Certainty of the evidence (GRADE)	Relative effect (95% CI)	Anticipated absolute effects	
				Risk with restrictive transfusion strategy	Risk difference with liberal transfusion strategy
Unfavorable neurological outcome (GOS 1-3)	2297 (4 RCTs)	⨁⨁⨁◯ Moderate[a]	**RR 0.88** (0.82 to 0.95)	606 per 1.000	**73 fewer per 1.000** (109 fewer to 30 fewer)
Unfavourable neurological outcome [DICHOTOMOUS]	2297 (4 RCTs)	⨁⨁⨁◯ Moderate[b]	**RR 0.88** (0.82 to 0.95)	553 per 1.000	**66 fewer per 1.000** (100 fewer to 28 fewer)
Overall mortality (until 12 months)	2385 (5 RCTs)	⨁⨁◯◯ Low[c]	**RR 0.97** (0.84 to 1.11)	251 per 1.000	**8 fewer per 1.000** (40 fewer to 28 more)

***The risk in the intervention group** (and its 95% confidence interval) is based on the assumed risk in the comparison group and the **relative effect** of the intervention (and its 95% CI).

CI: confidence interval; **RR:** risk ratio

GRADE Working Group grades of evidence
High certainty: we are very confident that the true effect lies close to that of the estimate of the effect.
Moderate certainty: we are moderately confident in the effect estimate: the true effect is likely to be close to the estimate of the effect, but there is a possibility that it is substantially different.
Low certainty: our confidence in the effect estimate is limited: the true effect may be substantially different from the estimate of the effect.
Very low certainty: we have very little confidence in the effect estimate: the true effect is likely to be substantially different from the estimate of effect.

Explanations

a. The confidence intervals for Gobatto 2019, HEMOTION and SAHARA, are wide and cross the line of no effect (RR = 1.0). Specifically, these intervals ([0.35, 1.29], [0.83, 1.03], and [0.78, 1.03]) include both potentially beneficial effects and the possibility of no effect. Downgraded by one level for imprecision.
b. The confidence intervals for Gobatto 2019, HEMOTION and SAHARA, are wide and cross the line of no effect (RR = 1.0), including both potentially beneficial effects and the possibility of no effect. Downgraded by one level for imprecision.
c. The confidence intervals for all the studies are notably wide and all of them cross the line of no effect (RR = 1.0). Therefore, the pooled estimate is affected by imprecision, warranting a downgrade of the evidence by two levels for imprecision.

References

1. Gaastra B, Ren D, Alexander S, et al. Evidence-based interconversion of the Glasgow Outcome and modified Rankin scales: pitfalls and best practices. J Stroke Cerebrovasc Dis. 2022;31(12):106845. doi:10.1016/j.jstrokecerebrovasdis.2022.106845

PRISMA 2020 Checklist

Section and Topic	Item #	Checklist item	Reported on page #
**TITLE**			
Title	1	Identify the report as a systematic review.	1
**ABSTRACT**			
Abstract	2	See the PRISMA 2020 for Abstracts checklist.	3
**INTRODUCTION**			
Rationale	3	Describe the rationale for the review in the context of existing knowledge.	5
Objectives	4	Provide an explicit statement of the objective(s) or question(s) the review addresses.	5
**METHODS**			
Eligibility criteria	5	Specify the inclusion and exclusion criteria for the review and how studies were grouped for the syntheses.	6
Information sources	6	Specify all databases, registers, websites, organisations, reference lists and other sources searched or consulted to identify studies. Specify the date when each source was last searched or consulted.	7
Search strategy	7	Present the full search strategies for all databases, registers and websites, including any filters and limits used.	7
Selection process	8	Specify the methods used to decide whether a study met the inclusion criteria of the review, including how many reviewers screened each record and each report retrieved, whether they worked independently, and if applicable, details of automation tools used in the process.	7,8
Data collection process	9	Specify the methods used to collect data from reports, including how many reviewers collected data from each report, whether they worked independently, any processes for obtaining or confirming data from study investigators, and if applicable, details of automation tools used in the process.	7,8
Data items	10a	List and define all outcomes for which data were sought. Specify whether all results that were compatible with each outcome domain in each study were sought (e.g. for all measures, time points, analyses), and if not, the methods used to decide which results to collect.	6,7
	10b	List and define all other variables for which data were sought (e.g. participant and intervention characteristics, funding sources). Describe any assumptions made about any missing or unclear information.	7,8
Study risk of bias assessment	11	Specify the methods used to assess risk of bias in the included studies, including details of the tool(s) used, how many reviewers assessed each study and whether they worked independently, and if applicable, details of automation tools used in the process.	8
Effect measures	12	Specify for each outcome the effect measure(s) (e.g. risk ratio, mean difference) used in the synthesis or presentation of results.	7,9
Synthesis methods	13a	Describe the processes used to decide which studies were eligible for each synthesis (e.g. tabulating the study intervention characteristics and comparing against the planned groups for each synthesis (item #5)).	6-8; Tab. 1
	13b	Describe any methods required to prepare the data for presentation or synthesis, such as handling of missing summary statistics, or data conversions.	7-8
	13c	Describe any methods used to tabulate or visually display results of individual studies and syntheses.	7-9
	13d	Describe any methods used to synthesize results and provide a rationale for the choice(s). If meta-analysis was performed, describe the model(s), method(s) to identify the presence and extent of statistical heterogeneity, and software package(s) used.	8,9
	13e	Describe any methods used to explore possible causes of heterogeneity among study results (e.g. subgroup analysis, meta-regression).	9
	13f	Describe any sensitivity analyses conducted to assess robustness of the synthesized results.	9
Reporting bias assessment	14	Describe any methods used to assess risk of bias due to missing results in a synthesis (arising from reporting biases).	8,9
Certainty assessment	15	Describe any methods used to assess certainty (or confidence) in the body of evidence for an outcome.	9

PRISMA 2020 Checklist

Section and Topic	Item #	Checklist item	Reported on page #
**RESULTS**			
Study selection	16a	Describe the results of the search and selection process, from the number of records identified in the search to the number of studies included in the review, ideally using a flow diagram.	9; Fig. 1
	16b	Cite studies that might appear to meet the inclusion criteria, but which were excluded, and explain why they were excluded.	9
Study characteristics	17	Cite each included study and present its characteristics.	9; Tab. 1
Risk of bias in studies	18	Present assessments of risk of bias for each included study.	10; eFigure 1.
Results of individual studies	19	For all outcomes, present, for each study: (a) summary statistics for each group (where appropriate) and (b) an effect estimate and its precision (e.g. confidence/credible interval), ideally using structured tables or plots.	10,11,12, supplement
Results of syntheses	20a	For each synthesis, briefly summarise the characteristics and risk of bias among contributing studies.	10,11,12, supplement
	20b	Present results of all statistical syntheses conducted. If meta-analysis was done, present for each the summary estimate and its precision (e.g. confidence/credible interval) and measures of statistical heterogeneity. If comparing groups, describe the direction of the effect.	10,11,12, supplement
	20c	Present results of all investigations of possible causes of heterogeneity among study results.	10,11,12, supplement
	20d	Present results of all sensitivity analyses conducted to assess the robustness of the synthesized results.	10
Reporting biases	21	Present assessments of risk of bias due to missing results (arising from reporting biases) for each synthesis assessed.	10,12, supplement
Certainty of evidence	22	Present assessments of certainty (or confidence) in the body of evidence for each outcome assessed.	12, supplement
**DISCUSSION**			
Discussion	23a	Provide a general interpretation of the results in the context of other evidence.	12-15
	23b	Discuss any limitations of the evidence included in the review.	12-15
	23c	Discuss any limitations of the review processes used.	12-15
	23d	Discuss implications of the results for practice, policy, and future research.	12-15
**OTHER INFORMATION**			
Registration and protocol	24a	Provide registration information for the review, including register name and registration number, or state that the review was not registered.	6
	24b	Indicate where the review protocol can be accessed, or state that a protocol was not prepared.	6
	24c	Describe and explain any amendments to information provided at registration or in the protocol.	7
Support	25	Describe sources of financial or non-financial support for the review, and the role of the funders or sponsors in the review.	N.A..
Competing interests	26	Declare any competing interests of review authors.	19.
Availability of data, code and other materials	27	Report which of the following are publicly available and where they can be found: template data collection forms; data extracted from included studies; data used for all analyses; analytic code; any other materials used in the review.	19.

PRISMA 2020 Checklist

From: Page MJ, McKenzie JE, Bossuyt PM, Boutron I, Hoffmann TC, Mulrow CD, et al. The PRISMA 2020 statement: an updated guideline for reporting systematic reviews. BMJ 2021;372:n71. doi: 10.1136/bmj.n71
